# PReS-FINAL-2054: Latent tuberculosis infection in patients with juvenile idiopathic arthritis undergoing methotrexate therapy: a longitudinal study with TST and ELISPOT

**DOI:** 10.1186/1546-0096-11-S2-P67

**Published:** 2013-12-05

**Authors:** F Sztajnbok, NL Boechat, SK Oliveira, SB Ribeiro, MC Rodrigues, C Diniz, FCDN Sztajnbok, CC Sant'Anna

**Affiliations:** 1 Pediatric Rheumatology, Universidade Do Estado Do Rio De Janeiro/Universidade Federal Do Rio De Janeiro, Brazil; 2 Pneumology, Universidade Federal Do Rio De Janeiro, Brazil; 3 Pediatric Rheumatology, Universidade Federal Do Rio De Janeiro, Brazil; 4 Pediatrics, Faculdade Técnico-Educacional Souza Marques, Brazil; 5 Pediatric Pneumology, Universidade Federal Do Rio De Janeiro, Rio De Janeiro, Brazil

## Introduction

Latent tuberculosis infection (LTBI) has a higher risk of developing the active form in immunosuppressed patients. Controversy exists regarding the accuracy of the tuberculin skin test (TST) and methods based on the production of interferon gamma in the diagnosis of TB in pediatric and immunosuppressed patients.

## Objectives

To contribute to the knowledge about the diagnosis of LTBI and TB disease in patients with juvenile idiopathic arthritis (JIA) treated with methotrexate in a high burden country, evaluating the frequency and evolution before and after its initiation, agreement between TST and ELISPOT and sensitivity and specificity of ELISPOT.

## Methods

This is an observational prospective longitudinal study where JIA patients starting methotrexate were evaluated in relation to clinical and epidemiological data, and TST and ELISPOT performed at inclusion and 3 and 12 months later.

## Results

There were a total of 24 patients. The prevalence of LTBI at inclusion was 20.8% (5/24 patients). The incidence of LTBI after initiation of immunosuppression was 26.3% (5/19 patients) and the prevalence of LTBI in the study as a whole was 41.6% (10/24 patients). The presence of epidemiological history positive for TB showed a relative risk of 2.0 for the development of LTBI. No patient developed TB disease. Only 2 patients had positive ELISPOT. Its sensitivity was 10%, specificity 92.8% and there was poor agreement between TST and ELISPOT.

## Conclusion

We found a high frequency of LTBI in patients with JIA and no superiority of ELISPOT compared to TST in the diagnosis and monitoring. The decision to start treatment for LTBI in immunosuppressed children and adolescents in a high burden country should be based on the results of TST, as the cost of ELISPOT is much higher and the accuracy is not superior to TST.

## Disclosure of interest

None declared.

